# The association of smoking status with healthcare utilisation, productivity loss and resulting costs: results from the population-based KORA F4 study

**DOI:** 10.1186/1472-6963-13-278

**Published:** 2013-07-17

**Authors:** Margarethe Wacker, Rolf Holle, Joachim Heinrich, Karl-Heinz Ladwig, Annette Peters, Reiner Leidl, Petra Menn

**Affiliations:** 1 Helmholtz Zentrum München - German Research Center for Environmental Health (GmbH), Institute of Health Economics and Health Care Management, Member of the German Center for Lung Research, Comprehensive Pneumology Center Munich (CPC-M), Ingolstaedter Landstr. 1, Neuherberg 85764, Germany; 2 Helmholtz Zentrum München - German Research Center for Environmental Health (GmbH), Institute of Epidemiology II, Member of the German Center for Cardiovascular Research, Ingolstaedter Landstr. 1, Neuherberg 85764, Germany; 3 Helmholtz Zentrum München - German Research Center for Environmental Health (GmbH), Institute of Epidemiology I, Member of the German Center for Lung Research, Comprehensive Pneumology Center Munich (CPC-M), Ingolstaedter Landstr. 1, Neuherberg 85764, Germany; 4 Ludwig-Maximilians-Universität München, Institute of Health Economics and Health Care Management, Ludwigstr. 28 RG, Munich 80539, Germany

**Keywords:** Smoking, Healthcare utilisation, Direct and indirect costs, Bottom-up approach, Germany

## Abstract

**Background:**

Smoking is seen as the most important single risk to health today, and is responsible for a high financial burden on healthcare systems and society. This population-based cross-sectional study compares healthcare utilisation, direct medical costs, and costs of productivity losses for different smoking groups: current smokers, former smokers, and never smokers.

**Methods:**

Using a bottom-up approach, data were taken from the German KORA F4 study (2006/2008) on self-reported healthcare utilisation and work absence due to illness for 3,071 adults aged 32-81 years. Unit costs from a societal perspective were applied to utilisation. Utilisation and resulting costs were compared across different smoking groups using generalised linear models to adjust for age, sex, education, alcohol consumption and physical activity.

**Results:**

Average annual total costs per survey participant were estimated as €3,844 [95% confidence interval: 3,447-4,233], and differed considerably between smoking groups with never smokers showing €3,237 [2,802-3,735] and former smokers causing €4,398 [3,796-5,058]. There was a positive effect of current and former smoking on the utilisation of healthcare services and on direct and indirect costs. Total annual costs were more than 20% higher (p<0.05) for current smokers and 35% higher (p<0.01) for former smokers compared with never smokers, which corresponds to annual excess costs of €743 and €1,108 per current and former smoker, respectively.

**Conclusions:**

Results indicate that excess costs for current and former smokers impose a large burden on society, and that previous top-down cost approaches produced lower estimates for the costs of care for smoking-related diseases. Efforts must be focused on prevention of smoking to achieve sustainable containment on behalf of the public interest.

## Background

Worldwide, tobacco smoking is seen as the most urgent health risk behaviour requiring prevention today [1]. The list of diseases caused by smoking includes cancer, cardiovascular diseases, respiratory illnesses and many more [2]. Smoking significantly reduces health-related quality of life [3,4] and is responsible for more than five million deaths worldwide every year [5]. Mortality and morbidity associated with smoking have considerable financial consequences for healthcare systems and economies [6,7]. In Germany, smoking is still prevalent despite a reduction in recent years: 30% of the population aged 18 and older are smokers, 26% former smokers, while 44% have never smoked [8]. Previous studies have shown that smoking results in higher utilisation of medical services [9,10], augmented work absenteeism [11-13] and increased resulting costs [14,15]. Despite their shortened life expectancy [16], smokers have higher lifetime medical costs [17-19].

Several studies have explored the financial burden of smoking in Germany [20-26], reporting total smoking-attributable costs from €17.4 billion to €33.6 billion for different base years, with productivity losses accounting for as much as between 72-74% of total costs [20,22]. However, these previous German studies all used a top-down approach. As this approach uses the concept of smoking-attributable fractions for mortality and morbidity known to be caused by smoking, the estimated financial burden of smoking may be inadequate if diseases and risks used for attribution do not fully reflect the structure of current smoking impact in Germany. For example, these studies used attributable mortality risks for attributing morbidity due to data limitations. Mortality risks may differ from morbidity risks and from the utilisation risk in the population at the time of sampling. Alternatively, the actual economic impact of smoking can be calculated by estimating excess costs of smokers compared to never smokers in a data set comprising current utilisation of individuals. This approach has the advantage of considering the entire spectrum of disease consequences associated with smoking and confounding factors [27].

The objective of this study was to assess differences in the utilisation of medical services and in direct and indirect costs from a societal perspective among current, occasional, former and never smokers using a population-based sample. To our knowledge, this is the first study in Germany to apply a bottom-up approach to estimate excess costs of smoking.

## Methods

### Data and study design

We used data from the KORA (Cooperative Health Research in the Augsburg Region) F4 survey, a population-based study conducted in 2006-2008 in Southern Germany. The F4 study is the follow-up of the KORA S4 survey (1999-2001). In brief, KORA S4 randomly selected 6,640 adults of German nationality aged 25-74 in the city of Augsburg and the two adjacent administrative districts from population registries; of those, 4,261 participated in the baseline examination. Details about design, sampling xmethod, data collection and response rates have been described elsewhere [28,29]. 3,080 individuals from S4 (72%) also participated in the follow-up survey F4, in which self-reported information on current smoking status, healthcare utilisation and non-productive working time was assessed in standardized computer-assisted interviews.

Participants were classified as current smokers if they smoked at least one cigarette per day at the time of the interview, as occasional smokers if they smoked usually less than one cigarette per day, as former smokers if they had smoked regularly or occasionally in the past and as never smokers if they had never smoked or less than 100 cigarettes in their lifetime.

Smoking status in F4 was compared with previous S4 information, and nine participants were excluded due to missing or implausible information on smoking status. Thus, the final study sample for this cross-sectional F4 analysis contained 3,071 subjects.

Information on age, sex, education (basic (≤9 years), secondary (10-11 years), higher (≥12 years)), alcohol consumption (based on WHO proceedings [30]: low risk alcohol consumption (average daily alcohol intake ≤ 20 g for women and ≤ 40 g for men), risky alcohol consumption (daily intake > 20 g for women and > 40 g for men)) and physical activity (active (regular sports in leisure time in summer and winter time for ≥ 1 hour per week), inactive (< 1 hour of sports per week)) were assessed in addition in F4.

A subsample of former smokers gave additional information on the date (n=1,164) and on reasons (n=791) for quitting in an additionally administered questionnaire.

### Assessment of healthcare utilisation and cost components

#### Utilisation of medical services

As described previously, participants were asked to state the number of times they had visited a physician in the previous 3 months, subdivided for 15 ambulatory specialisations [31]. Additionally, the numbers of ambulatory hospital visits, visits to alternative physicians and physical therapy treatments in the previous 12 months were recorded, as were the numbers of inpatient hospital days (including days in intensive care units), and inpatient and outpatient rehabilitations. Finally, use of pharmaceuticals in the previous week was assessed, detailing name, national drug code, dosage, interval of intake, and prescription status.

#### Direct costs

For monetary valuation of health services, national unit costs were applied as recommended by the Working Group Methods in Health Economic Evaluation (AG MEG) [32]. These unit costs were updated to the year 2008 (Additional file 1: Table S1).

Costs per physician contact for each medical specialty and per physical therapy unit were updated from 1999 to 2008 using the rate of change in physician reimbursement per case [33]. The resulting contact values for physician visits vary from €18.3 for psychiatrists to €99.6 for radiologists, and are €26.1 for physical therapy. The number of physician contacts and physical therapies was multiplied with the corresponding contact value to determine costs. If participants stated that they had visited a physician in the previous 3 months but did not indicate the number of visits (n=7), one visit was assumed following a conservative approach. In a sensitivity analysis, the mean number of visits was imputed instead.

Costs for alternative physicians were requested directly. If participants stated that they had visited an alternative physician but did not specify their costs (n=45), the average costs per visit stated by the users was imputed (€50).

As the reason for hospitalisation was unavailable, hospital days and days spent in the intensive care unit were valued using mean costs per day as suggested by the AG MEG. Unit costs were updated from 2000 to 2008 as described by the AG MEG and using data from the Federal Statistical Office [32,34,35], yielding costs of €451 per hospital day and €1,293 per day in the intensive care unit. Costs per ambulatory hospital treatment were assessed as one physician visit according to social legislation. Based on data on average costs and length of ambulatory and stationary rehabilitation from the German Pension Fund [36], costs per inpatient and outpatient rehabilitation were estimated as €100 and €62 per day, respectively.

Utilisation of pharmacy only pharmaceuticals was estimated using participant’s information about name, national drug code and dosage of intake within the previous week, and costs were calculated from 2008 pharmacy retail prices [37] by subtracting mandatory discounts of manufacturers and pharmacies according to the Social Security Code for statutory health insurance. Non-pharmacy medicines, dietary supplements and vitamins were excluded.

Total annual direct medical costs were calculated by extrapolating physician and drug costs to 12 months, and summing the costs of all health services.

#### Indirect costs

To assess indirect costs with regard to production losses in those aged 65 years or younger, participants were asked whether disability benefits were obtained, and those with regular employment were asked how many days they had been absent from work due to illness in the previous 12 months. If participants stated a greater number of days of absence from work than the maximum number of 213 working days in 2008 in Germany, their days of absenteeism were restricted to 213 (n=6).

Following current guidelines, we used the human capital approach to calculate productivity losses in paid work for society [38]. As methodological discussion about the most appropriate approach is still on-going, we additionally applied the frictional costs approach within a sensitivity analysis [39]. Whereas the human capital approach assumes a perfect labour market, the frictional costs approach charges only 80% of losses of the human capital approach to avoid potential overestimation of indirect costs [32].

Annual labour costs published by the Federal Statistical Office [40] were used to value productivity losses per year of disability in accordance with AG MEG guidelines. Costs per day of work lost were calculated by dividing annual labour costs by 213 working days in 2008, yielding costs of €160 per working day. Costs due to unpaid work, further premature retirement and death, as well as intangible cost resulting out of pain or decrease in quality of life, were not considered.

### Statistical analysis

Unadjusted analyses were performed regarding participants’ smoking status, healthcare utilisation and costs. To account for non-normality of cost data, 95% confidence intervals (CI) were estimated via a non-parametric bootstrap approach using a percentile method based on 1,000 replications.

The effect of smoking status on healthcare utilisation and work absenteeism was analysed in a two-step hurdle approach: In a first step we used multiple logistic regression models to calculate odds ratios of healthcare utilisation or inability to work as a function of smoking status, age, sex, education, alcohol consumption and physical activity. Secondly, for users only, factors influencing the number of healthcare service uses were analysed with generalised linear models assuming a zero-truncated negative binomial distribution with a log-link.

Finally, the effect of smoking status on direct, indirect and total healthcare costs was analysed, again adjusting for age, sex, education, alcohol consumption and physical activity. To consider the typically skewed distribution of costs, generalised linear models were used assuming a gamma distribution with log-link, where costs of €1 were assigned to participants with zero costs. This approach has been demonstrated to be a suitable method for healthcare costs [41,42].

Appropriate tests were operated to confirm the choice of distribution and link function. Regarding total costs, the Modified Park Test supported the choice of the gamma distribution (p=0.39), and the Hosmer-Lemeshow-Test (p=0.61), the Pregibon Link-Test (p=0.63) as well as the Pearson Correlation Test (p=0.90) all confirmed the choice of the log link function.

Due to missing data on three participants for education, the regression analysis contained 3,068 observations.

Statistical analyses were performed using SAS software (SAS Institute Inc., Cary, NC, USA, Version 9.2), and p-values of 0.05 or less were considered to be statistically significant.

Non-linear relationships of variables were checked as well as possible interactions by using variable selection methods (PROC GLMSELECT with stepwise selection method), but no significant non-linear relationships and interactions were observed.

## Results

### Unadjusted analyses

Table 1 gives the socio-demographic characteristics of the study sample. Of all participants, 15% were current smokers, 3% occasional smokers, 41% former smokers and 42% never smokers. Groups differ significantly regarding sex (p<0.0001), age (p<0.0001), and consequently in alcohol consumption (p<0.0001) and physical activity (p<0.01).

**Table 1:** Socio-demographic characteristics of the KORA F4 sample

		**Current smokers**	**Occasional smokers**	**Former smokers**	**Never smokers**	**Total**	**p-value**
		**n=468 (15.2%)**	**n=79 (2.6%)**	**n=1246 (40.6%)**	**n=1278 (41.6%)**	**n=3071 (100%)**	
**Sex**	Women	210 (44.9%)	41 (51.9%)	517 (41.5%)	824 (64.5%)	1592 (51.8%)	<0.0001 ^b^
	Men	258 (55.1%)	38 (48.1%)	729 (58.5%)	454 (33.5%)	1479 (48.2%)	
**Age**		49.68 (10.5)	48.84 (12.2)	57.40 (13.2)	57.49 (13.5)	56.04 (13.3)	<0.0001 ^c^
**Education**^**a**^	Basic education	237 (50.6%)	37 (47.4%)	648 (52.1%)	661 (51.8%)	1583 (51.6%)	0.32 ^b^
	Secondary education	134 (28.6%)	22 (28.2%)	301 (24.2%)	300 (23.5%)	757 (24.7%)	
	Higher education	97 (20.7%)	19 (24.4%)	296 (23.8%)	316 (24.8%)	728 (23.7%)	
**Alcohol consumption**	low risk (women: ≤20 g, men: ≤40 g alcohol per day)	382(81.6%)	69 (87.3%)	1031 (82.7%)	1160 (90.8%)	2642 (86.0%)	<0.0001 ^b^
	elevated risk (women: >20 g, men: >40 g alcohol per day)	86 (18.4%)	10 (12.7%)	215 (17.3%)	118 (9.2%)	429 (14.0%)	
**Physical activity**	active	228 (48.7%)	51 (64.4%)	699 (56.1%)	693 (54.2%)	1671 (54.4%)	0.01 ^b^
	inactive	240 (51.3%)	28 (35.4%)	547 (43.9%)	585 (45.8%)	1400 (45.6%)	

Data are presented as n (%)/mean (standard deviation). Any discrepancies in percentages are due to rounding.

^a^n=3 participants with missing information on school education. These subjects were excluded from the regression analysis.

^b^p-value based on Chi^2^-test.

^c^p-value based on ANOVA.

The proportion of users and the mean frequency of utilisation for each healthcare service are given in the Additional file 1: Table S1. Table S2 in the Additional file 1 shows average annual direct medical, indirect and total costs per smoking group. Eleven per cent of participants had zero total costs, 14% had no direct medical costs and 60% no indirect costs.

Overall, mean total annual costs per survey participant were €3,844 (95% CI: 3,847-4,233); €1,645 of these were indirect costs (43%), subdivided between costs of temporary absenteeism (43%) and disability benefits (57%). The largest shares of direct medical costs were due to hospital treatment (43%), pharmaceuticals (29%) and physician visits (15%).

Total costs ranked highest for former smokers at €4,398, followed by current smokers (€4,159), never smokers (€3,237) and occasional smokers (€3,074).

### Regression analysis

Table 2 reports the effect of smoking status on the probability of using healthcare services, being absent from work or receiving disability benefits. Current smokers showed significantly lower odds ratios for physician visits, physical therapy and work absence than never smokers. By contrast, former smokers showed significantly higher odds for hospital treatment, for rehabilitation and for pharmaceutical intake compared to never smokers.

**Table 2:** Probability of using medical services – results of logistic regression models – adjusted for age, sex, school education, alcohol consumption and physical activity

**Parameter**		**Physician visit**	**Hospital treatment**	**Rehabilitation**	**Physical therapy**^**a**^	**Alternative physician**	**Pharmaceuticals**	**Work absence**^**b**^	**Disability benefits**^**c**^
		***Odds ratio***	***Odds ratio***	***Odds ratio***	***Odds ratio***	***Odds ratio***	***Odds ratio***	***Odds ratio***	***Odds ratio***
		***[95% CI]***	***[95% CI]***	***[95% CI]***	***[95% CI]***	***[95% CI]***	***[95% CI]***	***[95% CI]***	***[95% CI]***
**Smoking status**	**Current smoker**	0.69 *** [0.55–0.87]	0.99 [0.73–1.33]	0.85 [0.48–1.49]	0.75 ** [0.58–0.97]	0.66 * [0.41–1.07]	1.20 [0.94–1.54]	0.75 ** [0.56–0.99]	1.41 [0.73–2.71]
	**Occasional smoker**	0.68 [0.42–1.09]	0.88 [0.46–1.71]	0.90 [0.27–2.96]	0.76 [0.44–1.31]	0.60 [0.21–1.68]	1.22 [0.73–2.05]	1.03 [0.57–1.85]	2.25 [0.64–7.87]
	**Former smoker**	1.12 [0.94–1.34]	1.24 ** [1.01–1.52]	1.47 ** [1.04–2.09]	1.00 [0.83–1.19]	1.21 [0.89–1.64]	1.39 *** [1.14–1.68]	1.19 [0.94–1.52]	1.56 * [0.92–2.65]
	**Never smoker**	1.00	1.00	1.00	1.00	1.00	1.00	1.00	1.00

n=3068 due to missing information on school education in three subjects.

*** significant at the 1% level/** significant at the 5% level/* trend with p≤0.10.

^a^n=3,067: four observations with missing information on physical therapy.

^b^n=1,499: work absence only for persons with information on occupational status and age ≤65.

^c^n=2,176: 22 observations with missing information on disability benefits.

The effect of smoking status on the number of medical services used and of working days absent is summarised in Table 3. Among users of physician treatments, current and former smokers showed higher numbers of visits than never smokers. Furthermore, former smokers used a greater amount of pharmaceuticals. Among participants who had been absent from work, the number of days absent from work was significantly higher in smokers.

**Table 3:** Frequencies of utilisation (users-only) – results of zero-truncated negative-binomial regression models – adjusted for age, sex, school education, alcohol consumption and physical activity

**Parameter**		**Number of physician visits**	**Number of hospital days (in-/outpatient)**	**Number of rehabilitation days**	**Number of physical therapies**	**Number of alternative physician visits**	**Number of pharmaceuticals used**	**Number of work absence days**
		**(n=1975)**	**(n=577)**	**(n=171)**	**(n=889)**	**(n=218)**	**(n=2172)**	**(n=796)**
		***exp(estimate)***	***exp(estimate)***	***exp(estimate)***	***exp(estimate)***	***exp(estimate)***	***exp(estimate)***	***exp(estimate)***
		***[95% CI]***	***[95% CI]***	***[95% CI]***	***[95% CI]***	***[95% CI]***	***[95% CI]***	***[95% CI]***
**Smoking status**	**Current smoker**	1.28 *** [1.08–1.52]	1.28 [0.74–2.19]	0.81 [0.59–1.11]	0.93 [0.75–1.15]	0.87 [0.43–1.77]	1.01 [0.88–1.16]	1.44 ** [1.03–2.01]
	**Occasional smoker**	1.00 [0.68–1.45]	0.83 [0.25–2.73]	1.18 [0.64–2.16]	0.84 [0.53–1.32]	0.36 [0.08–1.76]	1.33 * [1.00–1.76]	0.84 [0.45–1.55]
	**Former smoker**	1.17 *** [1.05–1.31]	0.89 [0.61–1.31]	0.91 [0.76–1.09]	1.14 * [0.99–1.31]	1.35 [0.86–2.11]	1.25 *** [1.14–1.39]	1.20 [0.93–1.54]
	**Never smoker**	1.00	1.00	1.00	1.00	1.00	1.00	1.00

*** significant at the 1% level/** significant at the 5% level/* trend with p≤0.10.

In all, smokers had a lower probability of physician treatments, but those utilising physician treatments showed an increased number of treatments compared with never smokers. Similarly, smokers showed a lower probability of being absent from work, but if they were absent the duration was 44% higher than in never smokers. In former smokers, both the probability of using pharmaceuticals and the number of pharmaceuticals used were greater compared to never smokers.

Table 4 shows the regression results for costs. Compared with never smokers, former smokers showed a 26% increase in direct medical costs (p=0.001) and 31% increase in indirect costs (p=0.03). Current and former smokers had 24% and 35% higher total annual costs (p=0.026 and p=0.001), respectively, than never smokers, corresponding to excess costs of €744 per current and €1,108 per former smoker.

**Table 4:** Annual direct medical, indirect and total costs – results of the Gamma regression models – adjusted for age, sex, school education, alcohol consumption and physical activity

**Parameter**		**Total direct medical costs**	**Total indirect costs**	**Total costs**
		***exp(estimate)***	***exp(estimate)***	***exp(estimate)***
		***[95% CI]***	***[95% CI]***	***[95% CI]***
**Smoking status**	**Current smoker**	1.06 [0.88–1.27]	1.28 [0.95–1.73]	1.24 ** [1.03 – 1.49]
	**Occasional smoker**	0.73 [0.50–1.06]	1.26 [0.68–2.36]	0.96 [0.65 – 1.41]
	**Former smoker**	1.26 *** [1.10–1.45]	1.31 ** [1.03–1.68]	1.35 *** [1.18 – 1.55]
	**Never smoker**	1.00	1.00	1.00

*** significant at the 1% level/** significant at the 5% level.

n=3,068 due to missing information on school education in three subjects.

1€ was assigned to observations with costs=0.

Additionally, age was associated with both higher direct and indirect costs, and men had higher indirect costs but lower direct medical costs than women. Participants with basic education had higher total annual costs. Risky alcohol consumption was associated with lowered direct medical costs. Participants who stated no regular physical activity showed higher direct costs than active participants. Detailed regression results can be found in the Additional file 1: Table S3.

Analysis of a subsample of former smokers showed that participants who quitted smoking in the last 12 months caused 2.37 (p<0.0005) times higher total costs than former smokers who quitted more than 12 months ago. Further subsample analysis in 791 former smokers showed that participants who quitted because of medical conditions caused significantly higher costs (92%) than former smoking participants who did not quit because of this reason. Former smokers who quitted in order to prevent future diseases showed only 63% of costs of participants for whom prevention was not an issue. Other reasons like financial aspects, pregnancy or fear of lung cancer had no significant influence on total costs in this subsample.

### Sensitivity analysis

Imputing the mean frequency of physician visits instead of one for the seven participants with missing frequencies did not affect results. Application of the frictional cost approach decreased indirect costs by 20% and total costs by 9%, but differences between smoking groups remained unchanged. Also, varying the amount of costs attributed to participants with zero costs in the regression analysis from €1 to €0.5 or €5 affected neither the coefficients nor their significance.

## Discussion

This study investigated the effect of smoking status on healthcare utilisation and direct and indirect costs in a population-based sample.

Total annual costs for current and former smokers were 24% and 35% higher, respectively, than for never smokers. Smoking was therefore associated with annual excess costs of €743 per current and €1,108 per former smoker. This was the first study to analyse costs of smoking in Germany using a bottom-up approach.

In all, our findings are in line with other international studies that have shown increased healthcare utilisation and costs in current and former smokers [6,9,12,14,15,43]. Similar to a health survey from the US [9], we found an increased risk of inpatient visits and an increased number of physician visits in former smokers. Our findings that current smokers showed a lower probability of physician treatments compared to never smokers, but a higher number of treatments if they used physician treatments at least once, is comparable to a recent study which found a decreased likelihood of current smokers to use primary care services but slightly increased costs [44]. This pattern could be explained by the possibility of a healthy smoker effect or special attitudes of smokers which translate into denial of disease and delays in seeking healthcare [44].

Regarding cost, our results suggest that former smokers incur even higher costs than current smokers. Within the subsample of former smokers, we observed that those who had quit smoking in the previous 12 months caused considerably higher total costs than those who had quit more than 12 months previously (factor 2.37, p=0.0005). Other studies also have found that former smokers induce higher medical costs within a period after cessation or shortly before quitting [9,14,45]. This may be explained by arising health problems being the reason for smoking cessation. Nevertheless, subsample analysis showed that even former smokers who had quit more than 10 years ago cause higher total annual costs than current smokers (28% vs. 23% higher total costs compared to never smokers, p=0.002).

Further analysis showed that subjects who quit smoking due to existing medical conditions had higher total costs irrespective of their period of non-smoking. We also found that former smokers who quit in order to prevent future diseases showed substantially lower total costs, which could be explained by overall more health-conscious behaviour.

Up to now, only a few studies have examined the economic burden of cigarette smoking in Germany [20-23]. Using a top-down approach, these studies identified direct and indirect costs attributable to smoking of €17.4 billion for the year 1993 [22] and €21 billion for the year 2003 [21]. As this approach uses the concept of smoking-attributable fractions for mortality and morbidity known to be caused by smoking, health conditions where smoking may be one of several contribution factors are neglected. Furthermore these previous top-down studies used attributable mortality risks for attributing morbidity. Mortality risks may differ from morbidity risks and from healthcare utilizations probabilities. In addition, in focussing on mortality risks this method solely considers smoking-related fatal diseases and neglects non-fatal health consequences of smoking like e.g. osteoporosis or eye diseases like cataract and glaucoma. Therefore this approach is known to downward-bias cost estimates [27].

Compared to top-down approaches, calculating excess costs based on subject-level data has the advantage of considering the entire spectrum of disease consequences associated with smoking. In addition, the bottom-up methodology used in this study provides better adjustment for the actual impact of smoking on health conditions and for population characteristics as it considers other differences in smokers and non-smokers regarding education and other risky behaviours besides smoking.

Disregarding issues of representativeness, the results of our study can be extrapolated to the whole population of Germany using data on national smoking prevalence [8]. Excluding costs of premature deaths, which were not measured in this study, smoking caused costs of €31.3 billion in 2008. Direct costs amounted to €17.9 as opposed to €8.23 billion in the top-down approach (inflated from €7.48 billion in 2003 using the consumer price index (CPI)) [21]. However, our results can only be based on the prevalence of smoking in a study population aged 32-81 years, which is lower than the national average used for projection. Taking these limitations into account, results strongly indicate that cost-of-illness estimates at the population level by the top-down approaches have been too conservative.

Compared with the most recent German top-down study, direct costs per current smoker derived from our bottom-up study exceed those of the top-down approach of €381 (inflated from €346 in 2003 using the CPI) [21] by nearly a factor of 2. This can be explained by the fact that top-down studies solely consider costs of diseases which are known to be associated with smoking or by different populations under study. Furthermore, using the bottom-up approach, costs for former smokers exceed those for current smokers by more than 45%. In contrast, using the top-down approach, costs for former smokers are smaller than those for current smokers, because all relative risks of smoking-related diseases for former smokers are smaller than those for current smokers. As former smokers in the cross-sectional KORA sample are about 8 years older than current smokers, they incur a higher probability of suffering from smoking-related disease with a delayed onset whereas health damage resulting of smoking may not yet have been revealed in current smokers.

A methodological issue, top-down studies base their calculation of excess cost on the entire adult population. In this bottom-up approach, the KORA population sample does not comprise younger age groups and might thus incur higher excess costs on average. As top-down studies do not consider age in the calculation of attributable healthcare utilisation and costs, an age-standardized comparison is beyond the scope of this paper. Differences in underlying age distribution are a restriction to this comparison of excess costs.

There are several limitations to our study. First, the cross-sectional design is susceptible to recall bias, as participants were asked to provide information retrospectively. By applying a recall time horizon of 1 week for pharmaceuticals, 3 months for physician visits and 12 months for more memorable services, the study design attempted to minimise this problem. This approach has been shown to be valid [46]. Nevertheless, recall error may have occurred, but it is unlikely to have affected the validity of our results because this may not have influenced the differences between the smoking groups [47].

Furthermore, as smoking habits were assessed by self-report without validation via biochemical tests, participants may have given socially desirable answers which are also prone to recall bias regarding smoking behaviour in the past. Therefore, the number of smokers may be underestimated, but any bias was minimised by comparing current information on smoking status with information from the previous survey.

Also, despite high recruitment efforts which positively affects representativeness [48], we cannot exclude selection bias. Whereas 75% of occasional, former and never smokers of the previous S4 survey were followed-up in the F4 survey, current smokers were underrepresented with a follow-up rate of 65%. This could be caused by higher death rates of current smokers or other unobserved factors, which could lead to systematic under- or overestimation of costs.

Monetary valuation of health services requires several assumptions that may cause under- or overestimation of costs. Also, unit costs were inflated to the year 2008. As the German healthcare sector has undergone considerable changes due to policy and law, real costs may have changed differently. Nevertheless, our updating approach represents a pragmatic approximation, and, although this may influence the amount of costs, differences between the smoking groups are unlikely to be affected.

Moreover, by using mean costs, differences in treatment intensity are not considered. Hospital costs in particular can differ significantly depending on the specialty department [32].

Drug costs were estimated based on participants’ information on intake and dosage. Using the defined daily dose methodology, as suggested by the WHO [49], leads to 5.4% higher costs with differences between smoking groups, but their ranking remains unchanged. As this approach assumes full compliance of patients, it was not considered.

Additional components of medical services such as nursing, medical aids and appliances and other medical therapies like logopaedia could not be included. However, the components considered in our study covered about 75% of total healthcare costs in 2008 [50]. Furthermore, direct non-medical costs, such as time and travel expenses, costs of premature death and intangible costs could not be considered. Although this probably led to underestimation of total costs for society, it is unclear if relative differences between smoking groups were affected.

Moreover, we cannot exclude confounding through differences in unobserved behaviours. The effect of smoking could, therefore, be under- or overestimated, for example by not adjusting for other risk behaviour patterns or socio-demographic characteristics, which could differ systematically between the smoking groups. In order to minimize potential confounding we controlled for alcohol consumption and physical activity. The inclusion of these factors hardly influenced the results concerning smoking status. Nevertheless, the levels of physical activity and alcohol consumption could also be the result of underlying health conditions which cannot be analysed in cross-sectional data. E.g. the finding that high risk alcohol consumption is associated with lower direct medical costs has to be explored in detail elsewhere.

Finally, the cross-sectional design of the KORA study gives a snapshot of medical utilisation and its costs, but does not allow longitudinal analysis using a life-cycle approach and therefore any accounting for the possibility of shorter life expectancy of smokers. Several studies have shown that lifetime medical costs of current and former smokers are increased compared with never smokers and outweigh potential cost savings due to shorter life expectancy [17-19].

## Conclusions

In conclusion, our results emphasise that smoking incurs high direct and indirect costs, and therefore imposes a significant burden on society. The economic impact of this public health problem highlights the importance of efforts aiming to discourage smoking. Although further research is needed to examine dose-effect relations of smoking, the timing of quitting on societal costs and results from longitudinal studies, policy makers should strengthen their efforts towards prevention, for example in realising the WHO Framework Convention on Tobacco Control [51] which suggests measures to reduce tobacco demand and therefore prevents smoking and its negative economic consequences.

## Competing interests

The authors declare no competing interests. The sponsors were not involved in the study design, collection, analysis and interpretation of data; in the writing of the manuscript; and in the decision to submit the manuscript.

## Authors’ contributions

MW conceptualised the paper, performed the statistical analysis, interpreted the data and drafted the manuscript. RH was involved in the coordination of the study and commented on drafts of the paper. RL commented on drafts of the paper. AP, JH and KHL were involved in the coordination of the study. PM supported conception, gave statistical support and assisted in writing the manuscript. All authors read and approved the final manuscript.

## Pre-publication history

The pre-publication history for this paper can be accessed here:

http://www.biomedcentral.com/1472-6963/13/278/prepub

## Supplementary Material

Additional file 1: Table S1 Utilisation of healthcare services and unit costs. **Table S2.** Unadjusted mean annual direct medical and indirect costs by smoking status. **Table S3.** Detailed results of regression analyses. Click here for file
