# Supplementary material for: The association of smoking status with healthcare utilisation, productivity loss and resulting costs: results from the population-based KORA F4 study
Source: BMC Health Serv Res. 2013 Jul 17;13:278. doi: 10.1186/1472-6963-13-278 (PMC3722023; doi:10.1186/1472-6963-13-278)
Supplement: Additional file 1: Table S1 — Utilisation of healthcare services and unit costs. Table S2. Unadjusted mean annual direct medical and indirect costs by smoking status. Table S3. Detailed results of regression analyses. [file 1472-6963-13-278-S1.doc]

**Additional file 1**

**Table S1: Utilisation of healthcare services and unit costs**

|  | | **Participants using resource** | | | **Frequency of utilisation  (if used)** | | | **Unit costs  per day / visit** |
| --- | --- | --- | --- | --- | --- | --- | --- | --- |
|  |  | n | % | | mean | std. | in € (2008) | |
| **Direct cost categories** | | | | | | | | |
| Physician visits in total (3 months) | | 1978 | 64.4 | | 4.39 | 4.75 |  | |
|  | General practitioner | 1381 | 45.0 | | 2.30 | 3.27 | 19.11 | |
|  | GP for internal medicine | 378 | 12.3 | | 2.31 | 2.06 | 22.20 | |
|  | Specialist for internal medicine | 240 | 7.8 | | 1.94 | 2.61 | 41.27 | |
|  | Gynaecologist | 315 | 10.3 | | 1.41 | 1.47 | 32.04 | |
|  | Ophthalmologist | 398 | 13.0 | | 1.47 | 1.38 | 31.18 | |
|  | Orthopaedist | 412 | 13.4 | | 2.21 | 1.99 | 27.93 | |
|  | Otorhinolaryngologist | 192 | 6.3 | | 1.84 | 2.16 | 29.65 | |
|  | Surgeon | 80 | 2.6 | | 2.30 | 2.77 | 27.53 | |
|  | Dermatologist | 224 | 7.3 | | 1.67 | 1.82 | 18.63 | |
|  | Radiologist | 225 | 7.3 | | 1.28 | 1.48 | 99.58 | |
|  | Urologist | 141 | 4.6 | | 1.48 | 0.95 | 34.43 | |
|  | Neurologist / psychiatrist | 148 | 4.8 | | 1.78 | 1.55 | 18.30 | |
|  | Psychotherapist | 57 | 1.9 | | 6.46 | 4.83 | 56.63 | |
|  | Specialist in occupational medicine | 51 | 1.7 | | 1.31 | 0.91 | 19.11 | |
|  | Other physician | 82 | 2.7 | | 1.52 | 1.35 | 27.44 | |
| Hospital treatment (12 months) | | | | | | | | |
|  | Inpatient hospital treatment | 426 | 13.9 | | 12.78 | 26.62 | 451.31 | |
|  | Intensive care unit | 64 | 2.1 | | 2.88 | 4.27 | 1292.94 | |
|  | Outpatient hospital treatment | 230 | 7.5 | | 2.31 | 3.98 | 27.44 | |
| Rehabilitation (12 months) | | | | | | | | |
|  | Inpatient rehabilitation | 118 | 3.8 | | 27.17 | 24.52 | 100.00 | |
|  | Outpatient rehabilitation | 53 | 1.7 | | 26.02 | 22.67 | 62.00 | |
| Physical therapy (12 months)^a^ | | 891 | 29.0 | | 17.92 | 20.99 | 26.09 | |
| Alternative physicians (12 months) | | 218 | 7.1 | | 5.26 | 7.58 | Self-reported costs | |
| Pharmaceuticals | | 2174 | 70.8 | | 3.16 | 2.43 | AOK Institute | |
| **Indirect cost categories^b^** | | | | | | | | |
| Work absence^c^ (number of days, 12 months) | | 796 | | 25.9 | 16.89 | 31.87 | 160.05 per day | |
| Disability benefits^d^ | | 85 | | 2.8 | - | - | 34,107 per year | |

^a^ n=3,067: 4 observations with missing information on physical therapy

^b^ n=2,198: indirect costs only for persons with employable age ≤ 65 years)

^c^ n=1,499: work absence only for full-time and regularly part-time employees

^d^ n=2,176: 22 observations with missing information on disability benefits

**Table S2: Unadjusted mean annual direct medical and indirect costs by smoking status (in 2008 € [95% CI]^a^)**

|  | **Current smokers** | **Occasional smokers** | **Former smokers** | **Never smokers** | **All** |
| --- | --- | --- | --- | --- | --- |
| ***Direct medical costs*** | | | | | |
| Physicians | 276 [218 – 349] | 241 [152 – 342] | 351 [319 – 383] | 312 [288 – 340] | 321 [303 – 339] |
| Hospital treatment | 995 [419 – 1795] | 439 [108 – 833] | 1025 [772 – 1372] | 906 [643 – 1219] | 956 [773 – 1162] |
| Rehabilitation | 77 [37 – 124] | 104 [0 – 235] | 156 [121 – 192] | 131 [87 – 186] | 132 [107 – 161] |
| Physical therapy^b^ | 92 [70 – 115] | 93 [47 – 148] | 148 [127 – 168] | 143 [123 – 164] | 136 [123 – 149] |
| Alternative physician | 24 [5 – 57] | 7 [1 – 16] | 26 [19 – 35] | 21 [14 – 28] | 23 [18 – 30] |
| Pharmaceuticals | 466 [349 – 562] | 383 [241 – 558] | 783 [544 – 1174] | 562 [470 – 665] | 632 [520 – 801] |
| **Total direct costs** | 1931 [1285– 2802] | 1266 [725 – 1826] | 2488 [2075 – 2985] | 2075 [1723 – 2465] | 2200 [1949 – 2486] |
| ***Indirect cost ^c^*** | | | | | |
| Work absence*^d^* | 989 [717 – 1333] | 513 [272 – 802] | 788 [621 – 961] | 512 [391- 649] | 701 [603 – 805] |
| Disability benefits^e^ | 1239 [728 – 1822] | 1295 [0 – 2590] | 1122 [766 – 1492] | 641 [400 – 894] | 944 [750– 1142] |
| **Total indirect costs** | 2220 [1610 – 2928] | 1808 [528 – 3406] | 1910 [1538 – 2298] | 1162 [892 – 1461] | 1645 [1432 – 1866] |
| **Total annual costs** | **4159 [3086 – 5284]** | **3074 [1612 – 4738]** | **4398 [3796 – 5058]** | **3237 [2802 – 3735]** | **3844 [3447 – 4233]** |

^a^ To account for non-normality of the cost data 95% confidence intervals (CIs) were estimated applying a non-parametric bootstrap approach using a percentile method with 1,000 replications.

^b^ n=3,067: 4 observations with missing information on physical therapy

^c^ n=2,198: indirect costs only for persons with employable age (≤ 65 years), mean costs calculated for all observations (n=3,071)

^d^ n=1,499: work absence only for persons with information on occupational status and age ≤ 65

^e^ n=2,176: 22 observations with missing information on disability benefits

**Table S3: Detailed results of regression analyses**

**Appendix to Table 2 in the main document: Probability of using medical services – results of logistic regression models**

| Parameter | | **Physician visit** | **Hospital treatment** | **Rehabilitation** | **Physical therapy^a^** | **Alternative physician** | **Pharma-ceuticals** | **Work absence^b^** | **Disability benefits^c^** |
| --- | --- | --- | --- | --- | --- | --- | --- | --- | --- |
|  |  | *Odds Ratio [95% CI]* | *Odds Ratio [95% CI]* | *Odds Ratio [95% CI]* | *Odds Ratio [95% CI]* | *Odds Ratio [95% CI]* | *Odds Ratio [95% CI]* | *Odds Ratio [95% CI]* | *Odds Ratio [95% CI]* |
| **Intercept** | | 0.32 ***  [0.22–0.46] | 0.08 ***  [0.05–0.13] | 0.01 ***  [0.01–0.03] | 0.38 ***  [0.26–0.56] | 0.37 ***  [0.19–0.72] | 0.13 ***  [0.08–0.19] | 3.69 ***  [1.92–7.12] | 0.0003 *** [0.00–0.003] |
| **Sex** | **Male** | 0.66 ***  [0.56–0.77] | 1.02  [0.84–1.23] | 0.94  [0.68–1.31] | 0.63 ***  [0.53–0.74] | 0.62 ***  [0.47–0.84] | 0.36 ***  [0.30–0.43] | 0.97  [0.79–1.19] | 1.68 **  [1.05–2.67] |
|  | **Female** | 1.00 | 1.00 | 1.00 | 1.00 | 1.00 | 1.00 | 1.00 | 1.00 |
| **Age** | | 1.03 ***  [1.03–1.04] | 1.02 ***  [1.01–1.03] | 1.02 ***  [1.01–1.03] | 1.01 **  [1.00–1.01] | 0.98 ***  [0.97–0.99] | 1.06 ***  [1.05–1.07] | 0.98 ***  [0.96–0.99] | 1.07 ***  [1.04–1.10] |
| **Smoking status** | **Current smoker** | 0.69 ***  [0.55–0.87] | 0.99  [0.73–1.33] | 0.85  [0.48–1.49] | 0.75 **  [0.58–0.97] | 0.66 *  [0.41–1.07] | 1.20  [0.94–1.54] | 0.75 **  [0.56–0.99] | 1.41  [0.73–2.71] |
|  | **Occasional smoker** | 0.68  [0.42–1.09] | 0.88  [0.46–1.71] | 0.90  [0.27–2.96] | 0.76  [0.44–1.31] | 0.60  [0.21–1.68] | 1.22  [0.73–2.05] | 1.03  [0.57–1.85] | 2.25  [0.64–7.87] |
|  | **Former smoker** | 1.12  [0.94–1.34] | 1.24 **  [1.01–1.52] | 1.47 **  [1.04–2.09] | 1.00  [0.83–1.19] | 1.21  [0.89–1.64] | 1.39 ***  [1.14–1.68] | 1.19  [0.94–1.52] | 1.56 *  [0.92–2.65] |
|  | **Never smoker** | 1.00 | 1.00 | 1.00 | 1.00 | 1.00 | 1.00 | 1.00 | 1.00 |
| **School education** | **Basic education** | 1.10  [0.90–1.33] | 1.06  [0.83–1.34] | 1.69 **  [1.07–2.66] | 1.13  [0.92–1.39] | 0.88  [0.62–1.25] | 1.02  [0.83–1.26] | 0.82  [0.65–1.05] | 1.90 **  [1.02–3.52] |
|  | **Secondary education** | 1.01  [0.81–1.26] | 0.92  [0.70–1.22] | 1.51  [0.91–2.52] | 1.01  [0.80–1.28] | 0.88  [0.60–1.29] | 1.12  [0.89–1.42] | 0.88  [0.67–1.14] | 1.25  [0.60–2.60] |
|  | **Higher education** | 1.00 | 1.00 | 1.00 | 1.00 | 1.00 | 1.00 | 1.00 | 1.00 |
| **Alcohol consumption** | **Elevated risk** | 0.91 [0.73–1.13] | 0.84 [0.64–1.11] | 1.25 [0.81–1.91] | 1.09 [0.87–1.37] | 1.15 [0.78–1.71] | 0.84 [0.67–1.07] | 1.00 [0.75-1.34] | 1.06 [0.60–1.89] |
|  | **Low risk** | 1.00 | 1.00 | 1.00 | 1.00 | 1.00 | 1.00 | 1.00 | 1.00 |
| **Physical activity** | **Inactive** | 1.17 ** [1.00–1.37] | 0.98 [0.81–1.18] | 0.84 [0.61–1.15] | 0.72 *** [0.62–0.85] | 0.69 ** [0.52–0.93] | 1.32*** [1.11–1.56] | 1.10 [0.90-1.36] | 1.26 [0.81–1.97] |
|  | **Active** | 1.00 | 1.00 | 1.00 | 1.00 | 1.00 | 1.00 | 1.00 | 1.00 |
| Deviance | | 3783.15 | 2928.01 | 1290.19 | 3623.48 | 1538.55 | 3253.20 | 2043.49 | 664.15 |

N=3068 due to missing information on school education in three subjects.

*** significant at the 1% level / ** significant at the 5% level / * trend with p≤0.10

^a^ n=3,067: four observations with missing information on physical therapy;

^b^ n=1,499: work absence only for persons with information on occupational status and age ≤65;

^c^ n=2,176: 22 observations with missing information on disability benefits.

**Appendix to Table 3 in the main document: Frequencies of utilisation (users-only) – results of zero-truncated negative-binomial regression models**

| **Parameter** | | **Number of physician visits** (n=1975) | **Number of hospital days (in-/outpatient)** (n=577) | **Number of rehabilitation days** (n=171) | **Number of physical therapies** (n=889) | **Number of alternative physician visits** (n=218) | **Number of pharmaceuticals used** (n=2172) | **Number of work absence days**  (n=796) |
| --- | --- | --- | --- | --- | --- | --- | --- | --- |
|  |  | *exp(estimate) [95% CI]* | *exp(estimate) [95% CI]* | *exp(estimate) [95% CI]* | *exp(estimate) [95% CI]* | *exp(estimate) [95% CI]* | *exp(estimate) [95% CI]* | *exp(estimate) [95% CI]* |
| **Intercept** | | 2.05 *** [1.58–2.64] | 1.82  [0.68–4.86] | 105.28 ***  [67.38–164.51] | 13.85 ***  [9.86–19.45] | 1.99  [0.65–6.08] | 0.29 *** [0.23–0.37] | 0.66  [0.32–1.37] |
| **Sex** | **Male** | 0.89 **  [0.80–0.99] | 0.89  [0.63–1.25] | 0.87  [0.74–1.03] | 0.91  [0.79–1.04] | 0.69  [0.44–1.10] | 0.91 **  [0.83–0.99] | 0.94  [0.75–1.17] |
|  | **Female** | 1.00 | 1.00 | 1.00 | 1.00 | 1.00 | 1.00 | 1.00 |
| **Age** | | 1.005 **  [1.00–1.01] | 1.01  [0.99–1.02] | 0.98 ***  [0.98–0.99] | 1.00  [1.00–1.01] | 1.01  [0.99–1.03] | 1.03 ***  [1.03–1.04] | 1.05 ***  [1.04–1.07] |
| **Smoking status** | **Current smoker** | 1.28 ***  [1.08–1.52] | 1.28  [0.74–2.19] | 0.81  [0.59–1.11] | 0.93  [0.75–1.15] | 0.87  [0.43–1.77] | 1.01  [0.88–1.16] | 1.44 ** [1.03–2.01] |
|  | **Occasional smoker** | 1.00  [0.68–1.45] | 0.83  [0.25–2.73] | 1.18  [0.64–2.16] | 0.84  [0.53–1.32] | 0.36  [0.08–1.76] | 1.33 *  [1.00–1.76] | 0.84  [0.45–1.55] |
|  | **Former smoker** | 1.17 ***  [1.05–1.31] | 0.89  [0.61–1.31] | 0.91  [0.76–1.09] | 1.14 * [0.99–1.31] | 1.35  [0.86–2.11] | 1.25 ***  [1.14–1.39] | 1.20  [0.93–1.54] |
|  | **Never smoker** | 1.00 | 1.00 | 1.00 | 1.00 | 1.00 | 1.00 | 1.00 |
| **School education** | **Basic education** | 1.20 ***  [1.05–1.38] | 1.10  [0.68–1.78] | 0.74 **  [0.58–0.94] | 1.07  [0.90–1.26] | 1.02  [0.59–1.77] | 1.09  [0.97–1.22] | 1.89 ***  [1.44–2.48] |
|  | **Secondary education** | 1.17 **  [1.00–1.37] | 1.51  [0.91–2.52] | 0.73 **  [0.55–0.96] | 0.96  [0.79–1.17] | 1.11  [0.62–1.98] | 1.03  [0.91–1.18] | 1.18  [0.89–1.55] |
|  | **Higher education** | 1.00 | 1.00 | 1.00 | 1.00 | 1.00 | 1.00 | 1.00 |
| **Alcohol consumption** | **Elevated risk** | 0.86 * [0.74–1.01] | 0.89 [0.54–1.47] | 1.03 [0.83–1.29] | 1.00 [0.83–1.20] | 1.42 [0.78–2.59] | 0.99 [0.87–1.11] | 1.02 [0.75–1.40] |
|  | **Low risk** | 1.00 | 1.00 | 1.00 | 1.00 | 1.00 | 1.00 | 1.00 |
| **Physical activity** | **Inactive** | 1.11 ** [1.00–1.23] | 1.16 [0.83–1.64] | 1.12 [0.95–1.33] | 0.85 ** [0.74–0.97] | 1.04  [0.68–1.59] | 1.13 *** [1.04–1.22] | 1.04 [0.83–1.30] |
|  | **Active** | 1.00 | 1.00 | 1.00 | 1.00 | 1.00 | 1.00 | 1.00 |
| Dispersion | | 1.0193 | 7.8988 | 0.2342 | 0.8776 | 2.1286 | 0.3864 | 2.7959 |

*** significant at the 1% level / ** significant at the 5% level / * trend with p≤0.10

**Appendix to Table 4 in the main document:**

**Annual direct medical, indirect and total costs – results of the Gamma regression models**

| Parameter | | **Total direct medical costs** | **Total indirect costs** | **Total costs** |
| --- | --- | --- | --- | --- |
|  |  | *exp(estimate)  [95% CI]* | *exp(estimate)  [95% CI]* | *exp(estimate)  [95% CI]* |
| **Intercept** | | 933.02 *** [705.83–1233.34] | 170.99 *** [90.13–324.40] | 3157.51 *** [2311.97–4312.30] |
| **Sex** | **Male** | 0.86 ** [0.76–0.97] | 1.44 *** [1.15–1.80] | 1.05 [0.93 – 1.20] |
|  | **Female** | 1.00 | 1.00 | 1.00 |
| **Age** | | 1.01 *** [1.01–1.02] | 1.04 *** [1.03–1.05] | 1.00 [0.99–1.00] |
| **Smoking status** | **Current smoker** | 1.06 [0.88–1.27] | 1.28  [0.95–1.73] | 1.24 ** [1.03 – 1.49] |
|  | **Occasional smoker** | 0.73  [0.50–1.06] | 1.26 [0.68–2.36] | 0.96 [0.65 – 1.41] |
|  | **Former smoker** | 1.26 *** [1.10–1.45] | 1.31 ** [1.03–1.68] | 1.35 *** [1.18 – 1.55] |
|  | **Never smoker** | 1.00 | 1.00 | 1.00 |
| **School education** | **Basic education** | 0.88 [0.75–1.04] | 1.49 *** [1.14–1.93] | 1.21 ** [1.03 – 1.41] |
|  | **Secondary education** | 0.98 [0.82–1.17] | 1.11 [0.83–1.47] | 1.07 [0.89 – 1.27] |
|  | **Higher education** | 1.00 | 1.00 | 1.00 |
| **Alcohol consumption** | **Elevated risk** | 0.79 *** [0.67–0.94] | 1.21 [0.90–1.63] | 0.98 [0.82–1.16] |
|  | **Low risk** | 1.00 | 1.00 | 1.00 |
| **Physical activity** | **Inactive** | 1.18 *** [1.04–1.33] | 1.15 [0.93–1.43] | 1.12 * [1.00–1.27] |
|  | **Active** | 1.00 | 1.00 | 1.00 |
| Scale parameter | | 1.44 [1.42–1.46] | 1.18 [1.17–1.18] | 1.43 [1.41–1.45] |
| Deviance | | 3.6632 | 9.4055 | 3.7804 |

*** significant at the 1% level / ** significant at the 5% level / * trend with p≤0.10

N=3,068 due to missing information on school education in three subjects.

1€ was assigned to observations with costs=0.
